# Is COVID-19 Associated With an Increased Risk of Subsequent Upper Respiratory Tract Infections in Adults? A Prospective Cohort Study

**DOI:** 10.1093/ofid/ofaf544

**Published:** 2025-09-02

**Authors:** Fazia Tadount, Guy Boivin, Yves Longtin, Patrice Savard, Matthew P Cheng, Hélène Decaluwe, Gaston De Serres, Élise Fortin, Caroline Quach

**Affiliations:** Centre Hospitalier Universitaire Sainte-Justine and Research Center, Montreal, Quebec, Canada; Department of Microbiology, Infectious Diseases, and Immunology, University of Montreal, Montreal, Quebec, Canada; Department of Microbiology-Immunology and Infectious Diseases, Laval University, Quebec City, Quebec, Canada; Infectious and Immune Diseases Axis, Research Center of the Centre Hospitalier de L’Université Laval, Quebec City, Quebec, Canada; Jewish General Hospital and Lady Davis Research Institute, Montreal, Quebec, Canada; Department of Microbiology, Infectious Diseases, and Immunology, University of Montreal, Montreal, Quebec, Canada; Immunopathology Axis, Research Center of the Centre Hospitalier de l’Université de Montréal, Montreal, Quebec, Canada; Infectious Disease Service, Centre Hospitalier de l’Université de Montréal, Montreal, Quebec, Canada; Divisions of Infectious Diseases and Medical Microbiology, McGill University Health Center, McGill University, Montreal, Quebec, Canada; McGill Interdisciplinary Initiative in Infection and Immunity, Montreal, Quebec, Canada; Centre Hospitalier Universitaire Sainte-Justine and Research Center, Montreal, Quebec, Canada; Department of Pediatrics, University of Montreal, Montreal, Quebec, Canada; Department of Social and Preventive Medicine, Laval University, Quebec City, Quebec, Canada; Department of Microbiology, Infectious Diseases, and Immunology, University of Montreal, Montreal, Quebec, Canada; Institut National de Santé Publique du Québec, Quebec City, Quebec, Canada; Centre Hospitalier Universitaire Sainte-Justine and Research Center, Montreal, Quebec, Canada; Department of Microbiology, Infectious Diseases, and Immunology, University of Montreal, Montreal, Quebec, Canada; Department of Pediatrics, University of Montreal, Montreal, Quebec, Canada

**Keywords:** COVID-19, epidemiology, upper respiratory tract infections

## Abstract

**Background:**

In Autumn 2022, a surge in upper respiratory tract infections (URTIs) was observed worldwide. Individuals anecdotally reported increased URTIs in the months following their coronavirus disease 2019 (COVID-19). The objective was to assess if COVID-19 is associated with a higher incidence of URTI in adults in the following months.

**Methods:**

**“**RECOVER” is a prospective cohort of health care workers (HCWs) from Montreal, Canada. HCWs completed biweekly surveys to report incident COVID-like symptoms. We included HCWs actively followed up for ≥90 days, between December 1, 2021, and December 31, 2022. Severe acute respiratory syndrome coronavirus 2 (SARS-CoV-2) infections were confirmed via reverse transcriptase polymerase chain reaction/antigenic testing. Non-COVID-19 URTI cases were defined as new onset of fever/sensation of fever, rhinitis, nasal congestion, sore throat/pharyngitis, sneezing, coughing, wheezing, difficulty breathing, increased respiratory secretions, or change in characteristics of chronic secretions, excluding symptoms within 48 hours of vaccination. Time-dependent Cox regression was used to assess the association between recent COVID-19 and URTI, adjusting for sex, age, workplace, household children <5 years, and asthma.

**Results:**

Among 320 HCWs (82.5% females; mean age, 42.4 years) followed for a median of 342 days, 152 (47.5%) participants tested positive for SARS-CoV-2. No significant difference in the incidence of URTI was observed following COVID-19 (hazard ratio, 1.03; 95% CI, 0.74–1.43; *P* = .87). However, having at least 1 child <5 years was associated with a 74% (95% CI, 20%–153%; *P* = .003) increase in the risk for URTI. Findings remained similar in sensitivity analysis.

**Conclusions:**

There was no association between COVID-19 and subsequent URTI. Other epidemiological, individual, and social factors could explain the increase in the incidence of URTI.

Upper respiratory infections not related to coronavirus disease 2019 (COVID-19) are widespread and associated with substantial morbidity, with up to 12.8 billion cases globally in 2021 [1]. During the first year of the COVID-19 pandemic, the activity of other respiratory viruses, such as influenza and respiratory syncytial virus (RSV), was substantially reduced and below historical levels [2, 3]. This was mainly due to nonpharmaceutical interventions (NPIs) implemented to mitigate severe acute respiratory syndrome coronavirus 2 (SARS-CoV-2) spread worldwide [3, 4]. However, in Autumn 2022, many countries experienced an important surge in upper respiratory tract infections (URTIs) [5, 6].

Questions on whether and how COVID-19 contributed to rates of other infections were raised [7]. While some referred to it as a consequence of a lack of immune stimulation due to the reduced exposure to a variety of pathogens following NPI deployment [7, 8], others hypothesized that SARS-CoV-2 infection could have long-lasting effects on the immune system, leaving some individuals more susceptible to infections [7, 9]. Recently, a retrospective cohort study of 228 940 children 0–5 years of age found that prior SARS-CoV-2 infection was associated with a significantly increased risk for RSV infection among young children in 2021 and 2022 [10]. Another hypothesis is that SARS-CoV-2 infection may disrupt the upper respiratory tract microbiome, potentially increasing susceptibility to other respiratory infections, although further research is needed to clarify this relationship [11–13].

Although a better understanding of the long-term effects of COVID-19 is crucial, epidemiological studies evaluating the association between COVID-19 and URTI remain scarce, particularly in adults. The Global Burden of Disease Study 2021 estimated the global, regional, and national burden of URTI, highlighting their significant public health impact, but did not address whether COVID-19 influences subsequent URTI risk [1]. Furthermore, recent data from Europe suggest age-related differences in postpandemic URTI trends, with the greatest increase in diagnoses among adults aged 18 to 30 years [14].

Despite these observations, prospective data on whether COVID-19 increases URTI risk in adults remain limited. To address this gap, we conducted a prospective cohort study to (1) assess if COVID-19 was associated with an increased risk of subsequent URTI in adults and (2) evaluate if exposure to COVID-19 would result in a higher recurrence of URTI episodes in adults. Our hypothesis was that prior COVID-19 infection is not associated with an increased risk of URTIs in adults.

## METHODS

This study followed the Strengthening the Reporting of Observational Studies in Epidemiology (STROBE) reporting guideline (Supplementary Data 1) [15].

### Study Design and Participants

As described elsewhere [16, 17], “RECOVER” is an observational prospective cohort study of health care workers (HCWs) who were enrolled following polymerase chain reaction (PCR)–confirmed SARS-CoV-2 infection to assess their risk of subsequent reinfection with the virus. Eligible HCWs comprised any professional working in the Greater Montreal (Quebec, Canada) area health care facilities [16]. A small group of participants—not necessarily HCWs, with no history of SARS-CoV-2 infection were also enrolled as controls (not previously infected or noninfected, as validated by negative serology). Participants were enrolled in the original RECOVER study between August 17, 2020, and November 16, 2021, and the last follow-up visit occurred on April 2, 2024.

During the first year of follow-up, participants presented to their local study site every 3 months, then every 6 months, and optional supplementary visits were scheduled upon SARS-CoV-2 reinfection/infection, as well as up to a week before and a month after receipt of a COVID-19 vaccine. Blood samples and demographic and clinical data were collected or updated at each visit. Furthermore, participants were actively followed up to monitor incident COVID-like symptoms. On a biweekly basis, each participant received an electronic questionnaire asking if they experienced new COVID-like symptoms during the past 14 days. Participants were required to report all incident symptoms, along with start date, and information on whether they were tested for COVID-19, along with test result, if applicable. Surveys were sent to participants’ email addresses and/or phone numbers, and a reminder to complete the survey was automatically sent after 48 hours (Supplementary Data 2). Notably, there was a temporary pause in survey distribution while awaiting ethics approval for a study extension, which affected some participants. Those who had completed their last scheduled follow-up before this administrative pause did not receive further questionnaires during that period.

The present analysis was conducted using all available longitudinal survey data and demographic information collected during the study. It focuses on a specific subperiod of RECOVER and is aimed at evaluating the relationship between prior COVID-19 infection and the risk of URTI. To be eligible for inclusion in this analysis, RECOVER participants had to be actively followed up for at least 90 days, starting December 1, 2021, up until December 31, 2022 (ie, study period).

### Exposure Definition

We defined exposure as a confirmed SARS-CoV-2 infection, based on standard clinical practice during the study period [18]. Both rapid antigenic tests and RT-PCR results were accepted for exposure ascertainment. Biweekly surveys had a reminder section telling participants experiencing COVID-like symptoms to get tested and to inform study site coordinators/nurses of the outcome.

### Outcome Definition

URTI cases were defined as experiencing at least 1 of the following symptoms: fever (≥38.0°C (≥100.4°F) or sensation of fever, rhinitis, nasal congestion, sore throat/pharyngitis, sneezing, coughing, wheezing, difficulty breathing, increased respiratory secretions, or change in characteristics of chronic secretions. This definition was adapted from the Canadian Nosocomial Infection Surveillance Program criteria for viral respiratory infections (VRIs), defined as onset of relevant symptoms, further confirmed with appropriate testing for respiratory pathogens [19]. Thus, our case definition was only symptom-based as laboratory testing was not possible in our study, except for COVID-19, for which HCWs were tested systematically if symptomatic.

An incident URTI episode was defined as the onset of new symptoms occurring ≥14 days after the start of a previous URTI episode. Any symptoms reported within 14 days of a prior episode were considered part of the same episode. Symptoms occurring within 2 days of vaccine receipt were excluded. Participants were allowed to add notes related to their symptoms in each biweekly survey. These notes were thoroughly reviewed by 3 independent reviewers (C.Q., É.F., and F.T.) to exclude any symptoms that were related to seasonal allergies, vaccination, or other irrelevant outcomes, which allowed for a more precise case definition. Fleiss’ kappa statistic was computed to evaluate the inter-rater reliability among reviewers.

### Statistical Analysis

We reported baseline characteristics of the cohort by COVID-19 exposure status. Measures of central tendency and variability were presented as means with SDs for normally distributed continuous variables and medians with interquartile ranges (IQRs) for non–normally distributed variables. Categorical variables were reported as proportions. Kaplan-Meier statistics were used to estimate the cumulative incidence of URTI episodes among exposed and nonexposed participants. COVID-19 exposure was set as a time-dependent variable to allow for change of status (ie, from nonexposed to exposed) during follow-up. In the main analysis, participants were defined as exposed if there was a COVID-19 episode during the study period through the end of follow-up. To assess the association between COVID-19 and risk of subsequent URTIs, multivariable Cox regression analyses with a time-dependent covariate (ie, COVID-19 status) were performed [20]. Crude hazard ratios (HRs) were computed and further adjusted for sex, age group (≤39, 40–49, and ≥50 years), workplace, the presence of at least 1 child under 5 years of age in the household, and underlying self-reported asthma (confirmed with medication use). Participants were followed until they were lost to follow-up, had a second COVID-19 episode, or if they reached the end of study follow-up, whichever occurred first. The Andersen-Gill recurrent event model, a generalization of the Cox proportional hazards regression that models the rate of URTIs over time, was used to evaluate the association between COVID-19 exposure and the recurrence of URTI episodes [21, 22]. To account for possible model misspecifications, we used robust variance estimates [23]. The model was adjusted for the same covariates as in the main analysis.

Finally, sensitivity analyses were carried out to assess the robustness of our findings. First, adjusted HRs for the association between COVID-19 and URTI were estimated using a 180-day risk window for exposure definition; that is, exposed participants were defined as such up to 6 months following a confirmed COVID-19 episode, after which they were considered no longer exposed. This time frame was chosen based on the biological plausibility that immunological dysfunction would potentially persist around 6 months following SARS-CoV-2 infection [24]. We further explored the presence of a differential misclassification bias to ensure that participants’ commitment to regularly fill out their biweekly questionnaires was not affected by their exposure status (eg, participants with a recent COVID-19 episode would be less keen on answering further questionnaires, which would result in an underreporting of the outcome in the exposed group). To assess for this bias, participants with low survey response rates (<50% during the study period) were removed from the analysis.

Furthermore, missing data on the presence of children were handled as a separate category, with missingness considered completely at random due to a technical issue that prevented this information from being recorded for a small subset of participants.

All reported *P* values are 2-sided with a significance level of .05. Statistical analyses were performed with RStudio, version 2023.6.1.524 [25], using the *survival* package [26]. Study data were collected and managed using Research Electronic Data Capture (REDCap) electronic data capture tools hosted at CHU Sainte-Justine Research Centre [27].

### Ethical Approval

The study was approved by the CHU Sainte-Justine Research Center Ethics Committee (Nagano platform project number MP-21–2021-3035). All participants provided written informed consent before enrollment.

## RESULTS

### Study Participants

**Figure 1.**
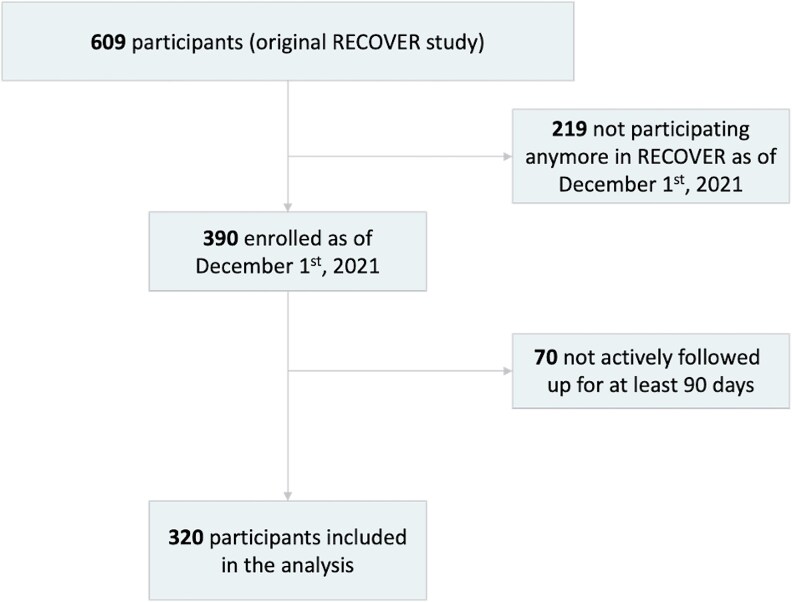
Flow diagram of study cohort.

The original RECOVER cohort comprised a total of 609 participants (ie, 570 with a history of infection and 39 noninfected controls—at enrollment), among whom 320 (52.5%) met eligibility criteria and were included in the present analysis (Figure 1). Participants were mostly females (82.5%) under 50 years of age (71.0%), with a mean age (SD) of 42.4 (11.1) years. They mainly worked at a health care facility (88.7%). The majority (91.9%) had a history of COVID-19 infection before the Omicron wave (ie, before December 1, 2021) and were vaccinated against COVID-19 (98.5%). Participants were generally healthy or had stable medical conditions, such as controlled hypertension, type 2 diabetes, or mild asthma (data not shown). The median duration of individual follow-up (IQR) was 342 (74.3) days, and a total of 152 (47.5%) had a confirmed SARS-CoV-2 infection during the study period (December 1, 2021, to December 31, 2022), whereas 168 (52.5%) did not report an infection. The proportion of participants who completed at least half of their biweekly questionnaires was 89.1%. Participants’ main demographic characteristics are shown in Table 1. Overall, both groups were comparable, despite a few notable differences in the proportions of exposed and nonexposed participants with regards to sex (134 [88.2%] vs 130 [77.4%] females), age (39.9 [10.2] vs 44.6 [11.5] years), and history of infection with SARS-CoV-2 before December 1, 2021 (134 [88.2%] vs 160 [95.2%]) (Table 1). Notably, none of the participants had reported a COVID-19 episode between March 15, 2021, and December 1, 2021 (ie, before the study period).

**Table 1.** Participant Characteristics at Baseline

Characteristic	Total (n = 320)	COVID-19 (+) (n = 152)	COVID-19 (−) (n = 168)
Sex (female)	264 (82.5)	134 (88.2)	130 (77.4)
Age, mean (SD), y	42.4 (11.1)	39.9 (10.2)	44.6 (11.5)
≤39 y	133 (41.6)	70 (46.1)	63 (37.5)
40–49 y	94 (29.4)	55 (36.2)	39 (23.2)
≥50 y	93 (29.1)	27 (17.8)	66 (39.3)
Ethnicity			
Caucasian	259 (80.9)	122 (80.3)	137 (80.3)
Asian	23 (7.2)	13 (8.6)	10 (6.0)
Black	19 (5.9)	11 (7.2)	8 (4.8)
Other^a^	19 (5.9)	6 (3.9)	13 (7.7)
Body mass index			
Underweight (BMI <18.5 kg/m^2^)	3 (0.9)	2 (1.3)	1 (0.6)
Normal (18.5 ≤ BMI < 25.0 kg/m^2^)	104 (32.5)	56 (36.8)	48 (28.6)
Overweight (25.0 ≤ BMI < 30.0 kg/m^2^)	100 (31.3)	51 (33.6)	49 (29.2)
Obese (BMI ≥30.0 kg/m^2^)	113 (35.3)	43 (28.3)	70 (41.7)
Comorbidities			
Allergies^b^	155 (48.4)	77 (50.7)	78 (46.4)
Asthma	38 (11.9)	17 (11.2)	21 (12.5)
Gastrointestinal	61 (19.1)	34 (22.4)	27 (16.1)
Heart/blood pressure	31 (9.7)	13 (8.6)	18 (10.7)
Group (history of COVID-19)^c^			
Infected	294 (91.9)	134 (88.2)	160 (95.2)
Noninfected	26 (8.1)	18 (11.8)	8 (4.8)
Workplace			
Acute care hospital	190 (59.4)	93 (61.2)	97 (57.7)
Public LTCF	67 (20.9)	26 (17.1)	41 (24.4)
Community health center	19 (5.9)	11 (7.2)	8 (4.8)
Private care facility	8 (2.5)	3 (2.0)	5 (3.0)
Other^d^	36 (11.3)	19 (12.5)	17 (10.1)
Overall follow-up time, median [range], d	342 [92–395]	353 [148–395]	335 [92–395]
Having at least 1 child age <5 y^e^	41 (21.1)	22 (14.5)	19 (11.3)
At least 1 dose of COVID-19 vaccine^f^	315 (98.4)	149 (98.0)	166 (98.8)
Influenza vaccine (2021–2022 season)^f^	79 (24.7)	40 (26.3)	39 (23.2)
Participants reporting at least 1 URTI^g^	116 (36.3)	61 (40.1)	55 (32.7)
1 URTI	67 (20.9)	33 (21.7)	34 (20.2)
2 URTIs	34 (10.6)	18 (11.8)	16 (9.5)
3 URTIs	10 (3.1)	6 (3.9)	4 (2.4)
4 URTIs	4 (1.3)	4 (2.6)	0 (0)
5 URTIs	1 (0.3)	0 (0)	1 (0.6)
Main symptoms reported for URTI episodes^h^			
Cough	97 (69.3)	49 (63.6)	48 (76.2)
Fever (≥38.0°C) or sensation of fever	34 (24.3)	19 (24.7)	15 (23.8)
Sore throat	114 (81.4)	62 (80.5)	52 (82.5)
Difficulty breathing	26 (18.6)	9 (11.7)	17 (27.0)
Nasal congestion, rhinorrhea	116 (82.9)	64 (83.1)	52 (82.5)
Completed biweekly surveys, mean (SD), %	66.8 (16.5)	66.5 (15.6)	67.1 (17.3)
≤50%	35 (10.9)	14 (9.2)	21 (12.5)
50%–70%	184 (57.5)	89 (58.6)	95 (56.5)
≥70%	101 (31.6)	49 (32.2)	52 (31.0)

Data are presented as No. (%) unless otherwise indicated.

Abbreviations: BMI, body mass index; COVID-19, coronavirus disease 2019; LTCF, long-term care facility; URTI, upper respiratory tract infection.

^a^Included Middle Eastern, Latin American, North American Aboriginal, or multiracial.

^b^Self-reported, physician-diagnosed environmental or food allergies.

^c^At least 1 confirmed COVID-19 episode before December 1, 2021.

^d^Included health care workers in private facilities, students, social/administrative workers in health care and other facilities.

^e^Missing information on the presence of children in the household for 13 (4.1%) participants.

^f^As of December 1, 2021.

^g^Total URTI episodes reported at the end of the study, regardless of the time sequence of COVID-19 exposure.

^h^Total reported symptoms across all URTI episodes (77 episodes in the exposed group and 63 in nonexposed. These values are used as denominators for the frequency of reported symptoms).

### Incidence of URTI

During the study period, 116 (36.3%) participants experienced at least 1 URTI episode, 61 (40.1%) in the COVID-19-exposed group and 55 (32.7%) in the nonexposed group (Table 1).

**Figure 2.**
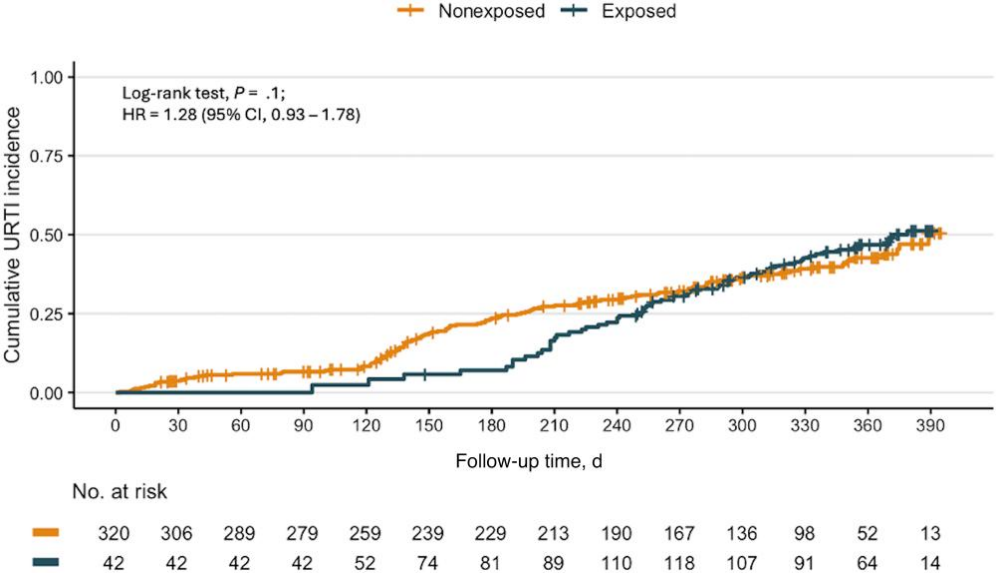
Unadjusted Kaplan-Meier estimates of overall URTI probability in COVID-19-exposed and nonexposed participants. Risk table shows participants at risk over time for both groups. Abbreviations: COVID-19, coronavirus disease 2019; URTI, upper respiratory tract infection.

**Figure 3.**
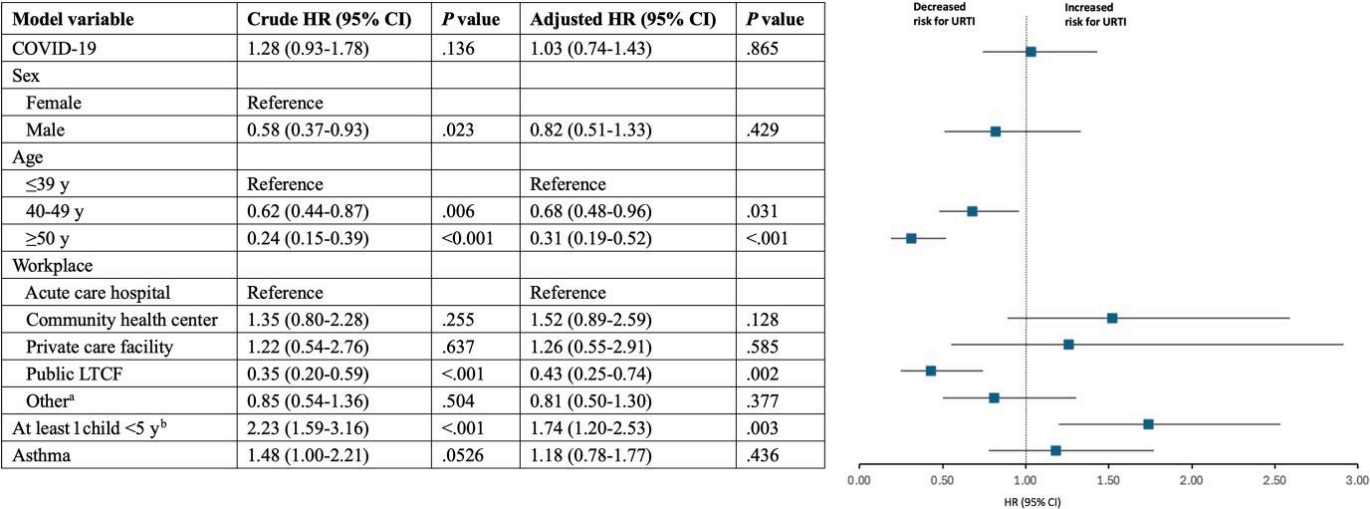
Multivariable-adjusted time-dependent Cox regression of URTI incidence following COVID-19 exposure. ^a^Included health care workers in private facilities, students, social/administrative workers in health care and other facilities. ^b^Missing data on the presence of children in the household (n = 13; 4.1%) were treated as a separate category in the analysis. Abbreviations: COVID-19, coronavirus disease 2019; HR, hazard ratio; URTI, upper respiratory tract infection.

The overall mean follow-up time until the first URTI episode was 321 days (95% CI, 301–341 days) in exposed and 303 days (95% CI, 289–317 days) in nonexposed participants. As shown in Figure 2, unadjusted Kaplan-Meier cumulative incidence curves showed no significant difference in the overall URTI incidence between groups (log-rank score test: *P* = .14; crude HR, 1.28; 95% CI, 0.93–1.78). After adjusting for confounders, recent COVID-19 episode was not associated with change in the risk of subsequent URTI (HR, 1.08; 95% CI, 0.77–1.51; *P* = .67). Interestingly, being 40 or 50 years old or older and working in a public long-term care facility (LTCF) were associated with significantly lower hazards for URTI (HR, 0.68; 95% CI, 0.48–0.96; *P* = .031; HR, 0.31; 95% CI, 0.19–0.52; *P* < .001; and HR, 0.43; 95% CI, 0.25–0.74; *P* = .002, respectively) (Figure 3). On the other hand, participants who reported having at least 1 child <5 years old in the household had a significantly higher risk for URTI (HR, 1.74; 95% CI, 1.20–2.53; *P* = .003) (Figure 3).

### Recurrence of URTI

The maximum number of URTI episodes for a participant during follow-up was 5, with 96% of all participants having at most 3 episodes. A total of 49 (15.3%) participants reported at least 1 URTI episode during their follow-up period (Table 1).

Results of the Andersen-Gill regression model for recurrent events are shown in Supplementary Table 1 (Supplement 3). Overall, older participants (age ≥50 years) and those working in a public LTCF had significantly lower risk of URTI recurrence (HR, 0.31; 95% CI, 0.18–0.54; *P* < .001; and HR, 0.43; 95% CI, 0.23–0.80; *P* = .008, respectively). However, participants who were living with young children, compared with those who were not, were more likely to have recurrent URTI episodes (HR, 1.74; 95% CI, 1.14–2.67; *P* = .011).

### Sensitivity Analyses

Using the 180-day exposure definition, results remained similar to what we reported in the main analysis; that is, COVID-19 was not associated with change in the risk of URTI. Conversely, being 40 or 50 years of age or older and working in a public LTCF were associated with a decreased risk of URTI, whereas having a child under 5 years of age was associated with increased risk (Supplementary Table 2 in Supplement 3). The same pattern was observed for regression of recurrent events (data not shown). We further applied a more stringent URTI definition, requiring at least 2 of the core symptoms listed in Table 1. The overall results were consistent with those observed in the primary analysis, except that both individuals 40 and 50 years of age and older showed a lower risk of URTI during follow-up (Supplementary Table 3 in Supplement 3). Finally, we assessed for the presence of a differential misclassification bias by excluding participants with low survey completion rates (<50%) from the analysis. Our findings remained unchanged, suggesting that adherence to filling out the biweekly questionnaires, and thus URTI outcome measurement, was unlikely to be influenced by recent COVID-19 in our cohort (Supplementary Table 4 in Supplement 3).

## DISCUSSION

We prospectively investigated the risk of URTI following SARS-CoV-2 infection in 320 HCWs during a 1-year follow-up. We found that a recent COVID-19 episode was not associated with an increased risk of subsequent URTI. Our findings suggest that factors other than prior SARS-CoV-2 infection, such as household and workplace exposures, play a more prominent role in influencing URTI risk among adults.

Specifically, we showed that having a young child (<5 years) in the household was associated with a 76% higher risk for URTI. Nevertheless, older individuals and workers in public LTCFs had about 60% lower risk of URTI. Finally, we assessed the overall effect of a recent COVID-19 exposure on the intensity of URTI recurrence and found lower rates in older participants (≥50 years) and those working in public LTCFs, whereas participants who reported having at least 1 child age <5 years in the household had higher rates of URTI recurrence. In sensitivity analyses, we used a 180-day COVID-19 exposure window and removed participants with low survey completion rates from the analysis, further confirming the robustness of our findings.

In Autumn 2022, many countries were hit by a surge of respiratory infections [5, 6]. Three viral epidemics: COVID-19, respiratory syncytial virus (RSV), and influenza, converged and created substantial pressure on health care systems [28]. The proportion of individuals reporting influenza-like illness (ILI) symptoms (ie, acute onset of cough and fever) during the 2022–2023 season in Canada was above historical levels from October to mid-December 2022 [29]. At that time, NPIs implemented during the pandemic had been widely relaxed. For instance, in the province of Quebec, those measures were completely lifted by June 2022, after a 2-month lockdown following the first Omicron detection in the province, at the end of November 2021 [30].

The surging numbers of people reporting respiratory illnesses raised questions on how the COVID-19 pandemic contributed to rates of other infectious diseases. The lack of immune stimulation due to reduced exposure to a variety of pathogens, secondary to NPI deployment (ie, immunity debt), was evoked as a possible explanation for this phenomenon [7, 8].

It was also hypothesized that SARS-CoV-2 itself may cause immune dysfunction, leaving some individuals more susceptible to infections [7, 9]. A recent study conducted in the United States showed that COVID-19 was associated with a significantly increased risk for RSV infections in children 0–5 years of age in 2021 and 2022 [10]. The authors suggested that the large buildup of COVID-19-infected children and the potential long-term adverse effects of COVID-19 on the immune and respiratory systems contributed to the 2022 surge of RSV cases in young children [10]. Additionally, emerging studies have proposed that SARS-CoV-2 may alter the upper respiratory tract microbiome, potentially affecting susceptibility to other infections [11–13].

However, epidemiological studies assessing the association between COVID-19 and respiratory infections in adults are scarce. Thus, we demonstrated that COVID-19 was not associated with a higher incidence of URTI in adults. Instead, individual, social, and epidemiological factors seemed to influence susceptibility to respiratory infections. We found that the presence of young children in the household was associated with an increased risk of URTI. Indeed, it was previously shown that preschool-aged household contacts are a risk factor for developing VRIs among HCWs working in outpatient settings [31]. We also found that age ≥50 years was associated with a lower risk of URTI. One plausible explanation is that older adults, particularly HCWs, tend to adhere more rigorously to infection prevention measures, including the use of personal protective equipment (PPE) [32]. This could lead to reduced exposure to respiratory pathogens. Finally, transmission of respiratory viruses is more likely to occur in certain health care settings compared with others [33]. In Quebec, LTCFs were severely impacted by the pandemic, especially during the first wave. Hence, several preventive measures were implemented to better protect LTCF residents who were elderly and frail [34]. Thus, in addition to decreasing SARS-CoV-2 transmission in LTCFs, those measures would have possibly contributed to reducing transmission of other respiratory viruses among both residents and facilities’ HCWs.

Our study has some important strengths. First, participants were prospectively followed and monitored biweekly for respiratory/COVID-like symptoms. The study design and active monitoring were likely to prevent recall bias in this cohort. Second, participants were mainly (>95%) HCWs, which makes outcome measurement reliable, as the precision in symptoms description was high and very clear for most participants. Furthermore, HCWs were systematically tested for COVID-19 upon symptom onset, which made exposure ascertainment more reliable. Nonetheless, participants who were eligible for this analysis were already enrolled in RECOVER for at least a year, since December 1, 2021, or earlier. Thus, they were familiar with study surveys, and completion rates were overall good and above >50% for most participants. Finally, the strong inter-rater reliability (κ = 0.89) observed in URTI case adjudication strengthens the internal validity of our study and minimizes the risk of misclassification bias in outcome definition.

### Limitations

Some limitations need to be considered in the interpretation of our results. As mentioned above, HCWs were generally tested for COVID-19 following symptom onset, meaning that asymptomatic cases were likely missed in our analysis. Additionally, URTI definition was only based on reported symptoms and could not be further confirmed as being a VRI (other than COVID-19). Moreover, our cohort is primarily composed of HCWs, who are predominantly females, Caucasians, and healthy or with medically stable comorbidities. Altogether, these factors hinder the generalizability of our findings to older and younger populations, people with comorbidities, and those of different ethnicities. However, the internal validity of our findings is unlikely to be compromised, as the cohort characteristics help reduce risk of residual and unmeasured confounding related to sex, age (elderly and children), comorbidities, and ethnicity.

Future research is needed to better understand the potential long-term effects of COVID-19 on immune function and susceptibility to other respiratory infections in adults. Expanding similar studies to non-HCW populations and more diverse cohorts would also help improve generalizability.

## CONCLUSIONS

In conclusion, we report no association between COVID-19 and the incidence of subsequent URTI. Other epidemiological, individual, and social factors—such as age, work setting, and the presence of young children in the household, could explain the increase in incidence of URTI in adults, especially HCWs.

## Supplementary Material

ofaf544_Supplementary_Data

## Supplementary Data

Supplementary materials are available at *Open Forum Infectious Diseases* online. Consisting of data provided by the authors to benefit the reader, the posted materials are not copyedited and are the sole responsibility of the authors, so questions or comments should be addressed to the corresponding authors.
