# Supplementary material for: Is COVID-19 Associated With an Increased Risk of Subsequent Upper Respiratory Tract Infections in Adults? A Prospective Cohort Study
Source: Open Forum Infect Dis. 2025 Sep 2;12(9):ofaf544. doi: 10.1093/ofid/ofaf544 (PMC12449730; doi:10.1093/ofid/ofaf544)
Supplement: ofaf544_Supplementary_Data [file ofaf544_supplementary_data.docx]

**Is COVID-19 Associated with an Increased Risk of Subsequent Upper Respiratory Tract Infections in Adults? A Prospective Cohort Study**

*(Supplemental content)*

**Supplement 1. STROBE Statement—checklist of items that should be included in reports of observational studies**

|  | Item No. | Recommendation | Page  No. | Relevant text from manuscript |
| --- | --- | --- | --- | --- |
| **Title and abstract** | 1 | (*a*) Indicate the study’s design with a commonly used term in the title or the abstract | 1 |  |
|  |  | (*b*) Provide in the abstract an informative and balanced summary of what was done and what was found | 2 |  |
| Introduction | | | |  |
| Background/rationale | 2 | Explain the scientific background and rationale for the investigation being reported | 3 | “However, in Autumn 2022, many countries experienced an important surge in upper respiratory tract infections (URTI).^5,6^ Questions on whether and how COVID-19 contributed to rates of other infections were raised.^7^” |
| Objectives | 3 | State specific objectives, including any prespecified hypotheses | 4 | “…we conducted a prospective cohort study to (1) assess if COVID-19 was associated with an increased risk of subsequent URTI in adults, and (2) evaluate if exposure to COVID-19 would result in a higher recurrence of URTI episodes, in adults. Our hypothesis was that prior COVID-19 infection is not associated with an increased risk of URTIs in adults.” |
| Methods | | | |  |
| Study design | 4 | Present key elements of study design early in the paper | 4 | “As described elsewhere,^16 17^ “RECOVER” is an observational prospective cohort study of healthcare workers (HCWs) that were enrolled following PCR-confirmed SARS-CoV-2 infection…” |
| Setting | 5 | Describe the setting, locations, and relevant dates, including periods of recruitment, exposure, follow-up, and data collection | 4-5 | “Eligible HCWs comprised any professional working in the Greater Montreal (Quebec, Canada) area healthcare facilities…”  “Participants were required to report all incident symptoms, along with start date, and information on whether they were tested for COVID-19, along with test result, if applicable.”  “We defined exposure as a confirmed SARS-CoV-2 infection, based on standard clinical practice during the study period” |
| Participants | 6 | (*a*) *Cohort study*—Give the eligibility criteria, and the sources and methods of selection of participants. Describe methods of follow-up | 5 | “Eligible HCWs comprised any professional working in the Greater Montreal (Quebec, Canada) area healthcare facilities… To be eligible for inclusion in this analysis, RECOVER participants had to be actively followed-up, for at least 90 days, starting December 1st, 2021, and up until December 31st, 2022 (i.e., study period).” |
|  |  | (*b*) *Cohort study*—For matched studies, give matching criteria and number of exposed and unexposed | NA |  |
| Variables | 7 | Clearly define all outcomes, exposures, predictors, potential confounders, and effect modifiers. Give diagnostic criteria, if applicable | 6-7 |  |
| Data sources/ measurement | 8* | For each variable of interest, give sources of data and details of methods of assessment (measurement). Describe comparability of assessment methods if there is more than one group | 5-6 |  |
| Bias | 9 | Describe any efforts to address potential sources of bias | 7-8 |  |
| Study size | 10 | Explain how the study size was arrived at | 5 | Figure 1 |

Continued on next page

| Quantitative variables | 11 | Explain how quantitative variables were handled in the analyses. If applicable, describe which groupings were chosen and why | 7-8 |  |
| --- | --- | --- | --- | --- |
| Statistical methods | 12 | (*a*) Describe all statistical methods, including those used to control for confounding | 6-8 |  |
|  |  | (*b*) Describe any methods used to examine subgroups and interactions | NA |  |
|  |  | (*c*) Explain how missing data were addressed | 7-8 |  |
|  |  | (*d*) *Cohort study*—If applicable, explain how loss to follow-up was addressed | NA |  |
|  |  | (*e*) Describe any sensitivity analyses | 7-8 |  |
| Results | | | | |
| Participants | 13* | (a) Report numbers of individuals at each stage of study—eg numbers potentially eligible, examined for eligibility, confirmed eligible, included in the study, completing follow-up, and analysed | 9 | Figure 1 |
|  |  | (b) Give reasons for non-participation at each stage | 9 | Figure 1 |
|  |  | (c) Consider use of a flow diagram |  | Figure 1 |
| Descriptive data | 14* | (a) Give characteristics of study participants (eg demographic, clinical, social) and information on exposures and potential confounders | 9-10 | Table 1 |
|  |  | (b) Indicate number of participants with missing data for each variable of interest |  | Table 1 and figure 3 |
|  |  | (c) *Cohort study*—Summarise follow-up time (eg, average and total amount) | 9 | Table 1 |
| Outcome data | 15* | *Cohort study*—Report numbers of outcome events or summary measures over time | 9-10 | Figure 2 |
| Main results | 16 | (*a*) Give unadjusted estimates and, if applicable, confounder-adjusted estimates and their precision (eg, 95% confidence interval). Make clear which confounders were adjusted for and why they were included |  | Figure 3 |
|  |  | (*b*) Report category boundaries when continuous variables were categorized |  | Table 1 |
|  |  | (*c*) If relevant, consider translating estimates of relative risk into absolute risk for a meaningful time period | NA |  |

Continued on next page

| Other analyses | 17 | Report other analyses done—eg analyses of subgroups and interactions, and sensitivity analyses | 10-11 |  |
| --- | --- | --- | --- | --- |
| Discussion | | | | |
| Key results | 18 | Summarise key results with reference to study objectives | 11 |  |
| Limitations | 19 | Discuss limitations of the study, taking into account sources of potential bias or imprecision. Discuss both direction and magnitude of any potential bias | 14 |  |
| Interpretation | 20 | Give a cautious overall interpretation of results considering objectives, limitations, multiplicity of analyses, results from similar studies, and other relevant evidence | 14 |  |
| Generalisability | 21 | Discuss the generalisability (external validity) of the study results | 14 |  |
| Other information | |  | | |
| Funding | 22 | Give the source of funding and the role of the funders for the present study and, if applicable, for the original study on which the present article is based | 15 |  |

*Give information separately for cases and controls in case-control studies and, if applicable, for exposed and unexposed groups in cohort and cross-sectional studies.

**Note:** An Explanation and Elaboration article discusses each checklist item and gives methodological background and published examples of transparent reporting. The STROBE checklist is best used in conjunction with this article (freely available on the Web sites of PLoS Medicine at http://www.plosmedicine.org/, Annals of Internal Medicine at http://www.annals.org/, and Epidemiology at http://www.epidem.com/). Information on the STROBE Initiative is available at www.strobe-statement.org.

**Supplement 2.** **Biweekly questionnaire used to track new COVID-like symptoms, for RECOVER cohort.**

Date of report: dd/MMM/yyyy

**SYMPTOMS**

- Did you experience any symptoms related to COVID-19, in the last 2 weeks?

❒ Yes ❒ No

*(If Yes)*

*Enter symptoms start date:* (dd/mmm/yyyy) : ___________

*Specify symptom(s) that you experienced:*

| Cough | ❒ Yes  ❒ No | Significant loss of appetite | ❒ Yes  ❒ No |
| --- | --- | --- | --- |
| Fever (≥38°C) or felt febrile | ❒ Yes  ❒ No | Loss sense of smell | ❒ Yes  ❒ No |
| Dizziness | ❒ Yes  ❒ No | Loss sense of taste | ❒ Yes  ❒ No |
| Difficulty breathing | ❒ Yes  ❒ No | Nasal congestion | ❒ Yes  ❒ No |
| Chest pain | ❒ Yes  ❒ No | Diarrhea | ❒ Yes  ❒ No |
| Fatigue | ❒ Yes  ❒ No | Nausea | ❒ Yes  ❒ No |
| Muscle aches | ❒ Yes  ❒ No | Vomitting | ❒ Yes  ❒ No |
| Sore throat | ❒ Yes  ❒ No | Abdominal pain | ❒ Yes  ❒ No |
| Headache | ❒ Yes  ❒ No |  |  |
| Other, specify: ________ | | | |

- Following these symptoms, have you been tested for COVID-19?

❒ Yes ❒ No

(If YES) What was the result of your test?

________________________

(If NO): ***Don’t forget to get tested if you think you had a significant exposure to a confirmed case or if your symptoms persist.***

**CONTACTS**

Did you have a significant contact (i.e. contact lasting more than 15 minutes within 2 meters) with known COVID-19 case(s) and without wearing the recommended personal protective equipment (PPE)?

❒ Yes ❒ No

**Additional comments/notes:**

________________________________________________________________________

|  |
| --- |
|  |
|  |
|  |

**REMINDER**

Do not forget to contact the research team (text message/phone) if:

1. A significant exposure to a COVID-19 case occurs in the workplace, without proper use of personal protective equipment (PPE).

2. If you have a nasopharyngeal swab taken at your occupational health and safety (OHS) office and the result is positive.

**Supplement 3. Sensitivity analyses**

**eTable 1**. Multivariable Adjusted Cox Regression for Recurrent Events (Andersen-Gill model)

| **Model variable** | **Adjusted HR (95% CI)** | ***P* value^a^** |
| --- | --- | --- |
| COVID-19 | 1.03 (0.72-1.47) | .87 |
| Sex (Male) | 0.82 (0.47-1.43) | .49 |
| Age |  |  |
| 18-40 y | Reference |  |
| 40-50 y | 0.68 (0.45-1.03) | .07 |
| ≥50 y | 0.31 (0.18-0.54) | <.001 |
| Workplace |  |  |
| Acute care hospital | Reference |  |
| Community health centre | 1.52 (0.80-2.88) | .20 |
| Private care facility | 1.26 (0.44-3.58) | .66 |
| Public LTCF | 0.43 (0.23-0.80) | .007 |
| Other | 0.81 (0.47-1.40) | .44 |
| At least one child <5 years^b^ | 1.74 (1.14-2.59) | .01 |
| Asthma | 1.18 (0.78-1.78) | .44 |

Abbreviations: LTCF, long-term care facility

^a^*P*-values were computed for the Andersen-Gill model with robust variance estimates.

^b^Missing information on the presence of children in the household for 13 (4.1%) participants.

**eTable 2.** Multivariable Adjusted Time-dependent Cox Regression of URTI Incidence following COVID-19 Exposure, using 180-day exposure definition.

| **Variable** | **Adjusted HR (95% CI)** | ***P* value** |
| --- | --- | --- |
| COVID-19 | 1.03 (0.74-1.44) | .87 |
| Sex |  |  |
| Female |  |  |
| Male | 0.85 (0.53-1.38) | .52 |
| Age |  |  |
| 18-40 y | Reference |  |
| 40-50 y | 0.67 (0.47-0.95) | .02 |
| ≥50 y | 0.31 (0.19-0.51) | .001 |
| Workplace |  |  |
| Acute care hospital | Reference |  |
| Community health centre | 1.58 (0.92-2.70) | .09 |
| Private care facility | 1.22 (0.53-2.81) | .64 |
| Public LTCF | 0.43 (0.25-0.74) | .002 |
| Other | 0.78 (0.48-1.25) | .30 |
| At least one child <5 years^a^ | 1.69 (1.19-2.39) | .003 |
| Asthma | 1.21 (0.80-1.82) | .36 |

^a^Missing information on the presence of children in the household for 13 (4.1%) participants.

**eTable 3.** Multivariable Adjusted Time-dependent Cox Regression of URTI Incidence following COVID-19 Exposure, with URTI Defined as Experiencing at least Two Symptoms.

| **Variable** | **Adjusted HR (95% CI)** | ***P* value** |
| --- | --- | --- |
| COVID-19 | 0.92 (0.62-1.35) | .65 |
| Sex |  |  |
| Female |  |  |
| Male | 0.75 (0.42-1.32) | .32 |
| Age |  |  |
| 18-40 y | Reference |  |
| 40-50 y | 0.53 (0.35-0.82) | .004 |
| ≥50 y | 0.33 (0.19-0.57) | <.001 |
| Workplace |  |  |
| Acute care hospital | Reference |  |
| Community health centre | 1.41 (0.76-2.62) | .27 |
| Private care facility | 0.61 (0.15-2.49) | .48 |
| Public LTCF | 0.41 (0.22-0.77) | .005 |
| Other | 0.84 (0.48-1.45) | .53 |
| At least one child <5 years^a^ | 1.60 (1.03-2.47) | .002 |
| Asthma | 1.12 (0.69-1.82) | .56 |

^a^Missing information on the presence of children in the household for 13 (4.1%) participants.

**eTable 4.** Multivariable Adjusted Time-dependent Cox Regression of URTI Incidence following COVID-19 Exposure, excluding participants with low survey completion rates (<50%).

| **Variable** | **Adjusted HR (95% CI)** | ***P* value** |
| --- | --- | --- |
| COVID-19 | 0.91 (0.64-1.29) | .60 |
| Sex |  |  |
| Female |  |  |
| Male | 0.78 (0.47-1.30) | .34 |
| Age |  |  |
| 18-40 y | Reference |  |
| 40-50 y | 0.68 (0.47-0.99) | .04 |
| ≥50 y | 0.31 (0.18-0.52) | <.05 |
| Workplace |  |  |
| Acute care hospital | Reference |  |
| Community health centre | 1.88 (0.85-2.57) | .16 |
| Private care facility | 0.55 (0.13-2.25) | .40 |
| Public LTCF | 0.39 (0.22-0.69) | .001 |
| Other | 0.75 (0.45-1.25) | .26 |
| At least one child <5 years^a^ | 1.79 (1.22-2.62) | .002 |
| Asthma | 1.14 (0.74-1.75) | .56 |

^a^Missing information on the presence of children in the household for 13 (4.1%) participants.
